# An examination of autism spectrum traits in adolescents with anorexia nervosa and their parents

**DOI:** 10.1186/2040-2392-5-56

**Published:** 2014-12-20

**Authors:** Charlotte Rhind, Elena Bonfioli, Rebecca Hibbs, Elizabeth Goddard, Pamela Macdonald, Simon Gowers, Ulrike Schmidt, Kate Tchanturia, Nadia Micali, Janet Treasure

**Affiliations:** Department of Psychological Medicine, Section of Eating Disorders, King’s College London, Institute of Psychiatry, Psychology and Neuroscience, PO59 103 Denmark Hill, London, SE5 8AF UK; Department of Public Health and Community Medicine, University of Verona, P.le L.A. Scuro 10, Verona, 37134 Italy; University of Liverpool, Psychological Sciences, Waterhouse Building, Block B, Brownlow Street, Liverpool, L69 3GL UK; Ilia State University, Kakutsa Cholokashvili Ave 3/5, Tbilisi, 0162 Georgia; Institute of Child Health, Behavioral and Brain Sciences Unit, University College London, 30 Guilford Street, London, WC1N 1EH UK

**Keywords:** Anorexia nervosa, Eating disorder, Autism spectrum disorder, Obsessive-compulsive disorder, Traits, Social aptitude, Adolescents, Parents, Development, Well-being assessment

## Abstract

**Background:**

There may be a link between anorexia nervosa and autism spectrum disorders. The aims of this study were to examine whether adolescents with anorexia nervosa have autism spectrum and/or obsessive-compulsive traits, how many would meet diagnostic criteria for autism spectrum disorder, and whether these traits are shared by parents.

**Methods:**

A total of 150 adolescents receiving outpatient treatment for anorexia nervosa or subthreshold anorexia nervosa and their parents completed the autism spectrum disorder and eating disorder sections of the Development and Well-being Assessment. Patients also completed the Children Yale-Brown Obsessive-Compulsive Scale and other measures of psychiatric morbidity, and parents completed the short Autism Quotient and Obsessive-Compulsive Inventory Revised.

**Results:**

Adolescents with anorexia nervosa had a below average social aptitude (19% below cut-off) and high levels of peer relationship problems (39% above cut-off) and obsessive-compulsive symptoms (56% above cut-off). Six cases (4%, all females) were assigned a possible (n = 5) or definite (n = 1) diagnosis of autism spectrum disorder. Parental levels of autism spectrum and obsessive-compulsive traits were within the normal range.

**Conclusions:**

This study suggests that adolescents with anorexia nervosa have elevated levels of autism spectrum traits, obsessive-compulsive symptoms, and a small proportion fulfil diagnostic criteria for a probable autism spectrum disorder. These traits did not appear to be familial. This comorbidity has been associated with a poorer prognosis. Therefore, adaptation of treatment for this subgroup may be warranted.

**Trial registration:**

Controlled-trials.com: ISRCTN83003225. Registered on 29 September 2011.

## Background

An association between low empathy during development and the later development of an eating disorder (ED) was suggested by Gillberg in 1992 [1]. He proposed that individuals with autism spectrum disorders (ASD) and those with anorexia nervosa (AN) may share traits, some of which might be familial [1]. Evidence summarized in recent reviews provides support for this hypothesis, suggesting that there may be a shared liability between AN and ASD [2–4]. For example, both disorders have a similar profile of neuropsychological function (problems with set shifting, central coherence, and social cognition) and behavioral traits such as social problems, and obsessive-compulsive features such as rigidity and attention to detail [2–4], although the neuropsychological profile of adolescents with AN is less clear (for an example see Lang *et al*. [5]).

Several studies have used the autism quotient (AQ), a self-report measure of autism spectrum traits, in people with AN and have found higher than normal scores [6, 7]. A study using the AQ and measures of systemizing and empathizing traits concluded that over 40% of adolescents with AN fulfilled the criteria for the broad autistic phenotype [8]. Another study found that 26% of adults with AN scored above the cut-off score on the short AQ [9]. In a large population study using parent report, 23% of children with restrictive eating problems also screened positive for ASD [10]. These studies suggest that there are shared traits between AN and ASD.

One problem in establishing the association between these conditions is that it can be difficult to make the diagnosis of ASD in women. Their clinical presentation differs from that typically seen in men, as summarized in a recent review [11]. Parents endorse different items for girls with ASD (such as ‘avoids demands’ , ‘very determined’ , and ‘careless with physical appearance and dress’) using a scale adapted to capture the female phenotype of ASD [12]. Furthermore, females present with more lifetime sensory problems [13]. It is thought that females with autism spectrum traits may be more likely to focus on eating or shape and weight issues as a topic of special interest.

People with EDs and their family members have higher levels of obsessive-compulsive personality traits [14, 15]. Behavioral traits, such as attention to detail and rigidity, are part of this personality profile and are common to those with EDs and ASD [2–4]. For example, the perceptual style of weak central coherence characterized by precedence for detail processing and/or difficulties with integrating global information is present in all three disorders [16–18]. A compulsivity phenotype may account for some of the common variance between these co-morbid conditions [19].

The aims of this study were to examine this dimensional approach to diagnosis by assessing whether adolescents with AN have autism spectrum and obsessive-compulsive traits, to establish the prevalence of ASD amongst this patient group, and whether these traits are shared by parents. It was hypothesized that (1) adolescents with AN would have higher levels of autism spectrum (poorer social aptitude skills and greater peer difficulties) and obsessive-compulsive traits, relative to population norms; (2) a higher proportion of adolescents with AN would fulfil diagnostic criteria for ASD than female healthy population norms, using the Development And Well-being Assessment (DAWBA) [20]; and (3) parents of adolescents with AN would have higher levels of autism spectrum and obsessive-compulsive traits, relative to population norms.

## Methods

### Participants

Adolescents newly referred for outpatient treatment with a primary diagnosis of AN or ED Not Otherwise Specified AN subtype (EDNOS-AN) and their parents completed a battery of assessments as part of a randomized controlled trial (Experienced Carers Helping Others (ECHO, trial registration: ISRCTN83003225)). Participants were recruited from 38 National Health Service (NHS) England outpatient services, providing an ecologically relevant sample representative of current United Kingdom practice. ED diagnoses were made by a clinician at the local recruitment site, according to the Diagnostic and Statistical Manual of Mental Disorders, 4th Edition (DSM-IV) criteria [21]. An age range of 13 to 21 years was chosen to represent an adolescent sample; up until brain development comes to completion (early twenties) [22] and usually whilst in full-time education. This is also consistent with previous similar adolescent trials [23]. Main ethics approval was granted by the Northwick Park Hospital Ethics Committee (approval number: 11/H0724/4) and site-specific ethics and governance approval granted on all participating sites (please see Acknowledgements). Written assent was collected for all patients and informed consent from their parents or guardians and all other participating carers was collected prior to entering the study in accordance with ethical guidelines. The methodology is described in detail elsewhere [24]. Only participants who completed the DAWBA and their parents were included in the present study. The final sample consisted of 150 adolescents (92% of total recruited) receiving treatment for AN (75%) or EDNOS-AN (25%) and their parents (n = 207). No differences were found between the participants included in the present study and the 163 patients and 224 parents recruited on clinical (body mass index (BMI), lowest BMI, and illness duration) and demographic variables (gender, employment, and marital status) with the exception of parental age which was greater in the sample included (data not shown).

### Patient measures

Standard demographic and clinical information were completed as self-report by patients (validated by clinicians) and their parents.

#### The developmental and well-being assessment

The DAWBA [20] is a well-validated and extensively used measure in epidemiological studies that is designed to generate a DSM-IV and an International Statistical Classification of Diseases and Related Health Problems (ICD-10) psychiatric diagnosis for childhood and adolescent psychiatric disorders. The ASD section is used as a diagnostic measure in population-based prevalence studies of ASD [25, 26]. In the present study, parents (informants) and patients (self-report) complete the ASD and ED sections of the computerized DAWBA which includes interview questions (each with screening questions) and skip rules. Parents report on their child’s development (language, routines, play, and social ability) and complete the Social Aptitudes Scale (SAS) [27], both designed to capture ‘traits’ rather than ‘state’ effects. The SAS is designed to tap the sorts of social aptitudes that require a good ability to read social and emotional cues rapidly in complex situations in order to guide socially skilled behaviour. Parents rate their child from ‘a lot worse than average’ to ‘a lot better than average’ , relative to other children of the same age, across 10 items. Low scores index a substantially raised risk of ASDs. The SAS has been well-validated and demonstrated good psychometric properties: data from a large epidemiological study of young people in the United Kingdom produced a mean score of 24.57 (SD = 6.26, n = 7768) with higher scores for females (mean = 25.33, SD = 6.14, n = 3764). A cut-off score of 16 or less is associated with good screening properties for the diagnosis of ASD [27]. Age-transformed aptitude scores with a mean of 50 and standard deviation of 10 have been developed [27]. Cronbach’s α coefficient (α) for responses to the SAS in the present study was 0.90.

The DAWBA generates six diagnostic probability bands (ranging from <0.1 to >70% likelihood) and a summary of structured and open-ended interview data, triangulated across informants by a trained experienced clinician (NM), who assigned a diagnosis (‘yes’ , ‘possible’ , or ‘no’) according to DSM-IV and ICD-10 criteria.

Parents and the individuals themselves also completed the Strengths and Difficulties Questionnaire (SDQ) [28] which consists of 25 items that assess behaviour problems, hyperactivity, emotional symptoms, peer problems, and pro-social skills. The sum of the first four subscale scores forms a total difficulties score, with higher scores indicating greater difficulties. Ratings of child distress and the impact of difficulties on social capital form a total impact score. Social skills can be inferred from the pro-social and peer relationship subscales. Good psychometric properties are reported for the SDQ. Normative data from a large epidemiological study [29] are available online with cut-off points; specifically, peer difficulties scores of 0 to 2 may be classified as normal, a score of 3 as ‘borderline’ , and scores of 4 to 10 as ‘abnormal’. In the current study, Cronbach’s α for responses to the SDQ symptom domains was 0.83 (informant) and 0.82 (self-report).

#### The children’s Yale-Brown obsessive-compulsive scale

Obsessive-compulsive traits in patients were assessed using the self-report Children’s Yale-Brown Obsessive-Compulsive Scale (CY-BOCS-SR) [30]. Higher scores indicate higher symptom severity (score range 0 to 40) and scores of 16 and over are used as a cut-off for clinically significant obsessive-compulsive disorder. Cronbach’s α for responses to the CY-BOCS-SR in the current study was 0.71.

#### The short evaluation of eating disorders

The Short Evaluation of EDs (SEED) [31] was completed by self-report to assess ED symptom severity. Responses are scored using an algorithm including weight and key symptoms and computed a total AN and Bulimia Nervosa (BN) symptom severity index (score range 0 to 3; higher score indicating severity). Cronbach’s α for the SEED in this study was 0.72.

#### Clinical impairment assessment 3.0

Psychosocial impairments related to the ED features over the past 28 days were also assessed using the Clinical Impairment Assessment (CIA) [32]. A global score is calculated with higher scores indicating greater severity of impairment. Cronbach’s α for the CIA in this study was 0.94.

#### The depression, stress and anxiety scale

The Depression, Stress and Anxiety Scale (DASS-21) [33] was used as a self-report measure of current mood state. Cronbach’s α for the DASS-21 was 0.94.

### Parent measures

#### The short autism quotient

Autism spectrum traits in parents were assessed using the Short Autism Quotient (AQ-10) [34]. Healthy adults produced a mean score of 2.77 (SD = 2.00) and a cut-off point of 6 is indicative of ASD [34]. Cronbach’s α for the AQ-10 in this study was 0.56.

#### The obsessive-compulsive inventory

Obsessive-compulsive traits in parents were measured using the Obsessive-Compulsive Inventory (OCI-R) [35]. A cut-off point of 21 is indicative of clinically significant obsessive-compulsive disorder. Cronbach’s α for the OCI-R in this study was 0.88.

### Data analysis

Data were analyzed using IBM SPSS Statistics for Windows, Version 21.0: Armonk, New York. Descriptive statistics summarized means and standard deviations for patient and parent scores on all measures. Independent samples t-tests were used for group comparisons: social aptitude scores in present study sample versus population mean; patients who did versus did not receive a diagnosis for ASD; and mothers versus fathers. Cohen’s d was used to calculate effect sizes. Spearman’s correlations were used to measure associations between patients autism spectrum and obsessive-compulsive traits, and their current ED clinical characteristics, and parental traits, applying a Bonferroni-corrected α (0.05 out of 13, α = 0.004) to correct for multiple comparisons.

## Results

The patient sample was 91% female, 96% Caucasian, and 91% were living with their parents. The age ranged from 13 to 21 years (mean = 16.90, SD = 2.13) and 83.6% were within three years of illness onset. The majority of patients (69%) had received no prior treatment for their ED. One patient in the sample reported having received a diagnosis of ASD. Other clinical information is displayed in Table 1. The mother (mean age = 48 years, SD = 4.82) was involved in the study for 95% of patients and the father for 42% (mean age = 51 years, SD = 5.06).

**Table 1:** **Sociodemographic and clinical characteristics**

	Females(N = 137)Mean(SD,range)/n(valid %)	Males(N = 13)Mean (SD, range)/n(valid %)	Total(N = 150)Mean(SD,range)/n(valid %)	ASD subset(N = 6, all females)Mean(SD,range)/n(valid %)
Age (years)	16.86 (2.11, 12.42-21.57)	17.31 (2.37, 13.20-21.53)	16.90 (2.13, 12.42-21.57)	17.09 (1.95, 15.27-20.21)
Illness duration (months)	22.83 (23.60, 2.00-110.00)	20.58 (11.26, 4.00-39.00)	22.64 (22.79, 2.00-110.00)	19.00 (15.45, 4.00-49.00)
BMI	16.81 (2.17, 12.38-24.20)	17.16 (2.44, 13.00-21.60)	16.84 (2.19, 12.38-24.20)	16.45 (2.25, 14.30-20.50)
Weight-for-height (%)^a^	82.50 (11.58, 55.07-122.97)	84.77 (15.78, 65.19-106.14)	82.65 (11.82, 55.07-122.97)	79.93 (10.94, 65.98-92.70)
Lowest BMI	15.45 (2.29, 11.40-23.60)	15.22 (2.63, 12.00-18.30)	15.43 (2.30, 11.40-23.60)	14.56 (1.20, 13.10-16.40)
Previous hospital admission				
0	120 (92.3%)	11 (91.7%)	131 (92.3%)	5 (100%)
1	9 (6.9%)	1 (8.3%)	10 (7.0%)	0
2	1 (0.8%)	0	1 (0.7%)	0
Missing	7 (5.1%)	1 (7.7%)	8 (5.3%)	1 (16.7%)
DAWBA ED diagnosis^b^				
AN	82 (59.9%)	5 (38.5%)	87 (58.0%)	3 (50.0%)
EDNOS	49 (35.8%)	7 (53.8%)	56 (37.3%)	3 (50.0%)
BN	1 (0.7%)	0	1 (0.7%)	0
Possible	1 (0.7%)	0	1 (0.7%)	0
No	4 (2.9%)	1 (7.7%)	5 (3.3%)	0

^a^Age standardized weight-for-height (participants aged <20 years (n = 126) only are reported), according to Great Ormond Street Hospital for Children criteria.

^b^Clinical rating using the DAWBA (DSM-IV and ICD-10 criteria).

Abbreviations: AN, Anorexia Nervosa; ASD, Autism spectrum disorder; BMI, Body Mass Index; BN, Bulimia Nervosa; DAWBA, Development and Well-being Assessment; ED, Eating Disorder; EDNOS, Eating Disorder Not Otherwise Specified.

### Psychometric assessment of adolescents with anorexia nervosa

Social aptitude scores (mean = 22.87, SD = 8.16) were lower than the healthy population norm (see legend, Table 2; t = 3.03, *p* = 0.002, d = -0.23), particularly for females (mean = 22.71, SD = 8.35; t = 4.51, *p* <0.001, d = -0.36). Scores fell within the clinically significant range for 18.8% of patients. They were not significantly correlated with clinical characteristics (BMI, illness duration, AN symptom severity, BN symptom severity, ED clinical impairment, depression, anxiety, or stress scores; data not shown).

**Table 2:** **Patient psychiatric symptomatology and comorbidity**

	All females(N = 137)Mean(SD)	All males (N = 13)Mean(SD)	Total(N = 150)Mean(SD)	ASD subset(N = 6,all females)Mean(SD)
CY-BOCS total	16.95 (8.79)	13.58 (8.34)	16.63 (8.77)	16.00 (9.82)
SAS^a^	22.71 (8.35)	24.70 (5.29)	22.87 (8.16)	7.00 (2.37)
SAS T-score^a^	45.50 (12.01)	48.00 (7.23)	45.70 (11.69)	23.50 (2.34)
SDQ Peer problems^b^				
Self-report	3.14 (2.05)	3.00 (1.55)	3.13 (2.01)	4.60 (2.88)
Informant	2.95 (2.19)	3.36 (2.16)	2.98 (2.18)	5.67 (1.75)
SDQ Pro-social skills^c^				
Self-report	7.93 (1.70)	7.82 (1.66)	7.92 (1.69)	7.20 (0.84)
Informant	7.08 (2.29)	7.64 (2.20)	7.13 (2.28)	4.50 (1.98)
SDQ Emotional				
Self-report	6.63 (2.35)	6.91 (2.34)	6.65 (2.34)	6.40 (2.97)
Informant	6.59 (2.45)	5.82 (2.27)	6.52 (2.44)	8.00 (1.10)
SDQ Conduct				
Self-report	2.34 (1.70)	1.73 (1.35)	2.29 (1.68)	2.80 (1.64)
Informant	2.42 (1.74)	2.27 (2.37)	2.41 (1.79)	3.00 (2.37)
SDQ Hyperactivity				
Self-report	5.14 (2.29)	5.27 (1.95)	5.16 (2.16)	5.80 (2.39)
Informant	4.00 (2.30)	3.64 (1.86)	3.97 (2.26)	4.67 (2.51)
SDQ Total difficulties				
Self-report	17.24 (6.17)	16.91 (4.66)	17.22 (6.04)	19.60 (7.13)
Informant	15.96 (6.48)	15.09 (6.28)	15.89 (6.44)	21.33 (5.50)
SDQ Impact				
Self-report	4.40 (3.36)	3.73 (4.65)	4.34 (3.46)	4.40 (3.65)
Informant	3.99 (3.14)	3.82 (3.25)	3.98 (3.14)	7.17 (0.75)
ASD probability band^d,e^				
<0.1%	102 (85.7%)	10 (91.9%)	112 (86.2%)	1 (16.7%)
Approx. ~ 3%	13 (10.9%)	1 (9.1%)	14 (10.8%)	4 (66.7%)
Approx. 15%	3 (2.5%)	0	3 (2.3%)	1 (16.7%)
Approx. 50%	1 (0.8%)	0	1 (0.8%)	0
Missing	18 (13.1%)	2 (15.4%)	20 (13.3%)	0
Diagnosis ASD^e^				
Possible	5 (3.6%)	0	5 (3.3%)	5 (83.3%)
Yes	1 (0.7%)	0	1 (0.7%)	1 (16.7%)

^a^Lower scores indicate poor social aptitude (cut-off score 16 or less). British (age 11 to 14 years) healthy population mean = 24.57 (SD = 6.26), females only mean = 25.33 (SD = 6.14), males only mean = 23.81 (SD = 6.38). SAS Age-Transformed-score mean = 50 (SD = 10) [27].

^b^British (age 11 to 14 years) healthy population informant-rated mean = 1.5 (SD = 1.7), self-report mean = 1.5 (SD = 1.4) [29].

^c^British (age 11 to 14 years) healthy population informant-rated mean = 8.6 (SD = 1.6), self-report mean = 8.0 (SD = 1.7) [29].

^d^British (age 11 to 14 years) healthy population females (F) and males (M) selecting those with no intellectual disability ASD band <0.1% = 98.3% (F), 95.7% (M); ASD band approximately 3% = 1.2% (F), 2.1% (M); ASD band approximately 15% = 0.3% (F), 1.4% (M); ASD band approximately 50% = 0.2% (F), 0.4% (M).

^e^Valid percentages presented.

^e^Clinical rating using the DAWBA (DSM-IV and ICD-10 criteria).

Abbreviations: SAS, Social Aptitude Scale; SDQ, Strengths and Difficulties Questionnaire; ASD, Autism Spectrum Disorder.

For both categories of rater (informant (I) and self-report (SR)) completing the SDQ, the highest levels of difficulties relative to norms were in the emotional (I: d = 2.07, SR: d = 1.73) and peer domains (I: d = 0.76, SR: d = 0.94). Peer difficulties were ‘abnormal’ for 38.5% of patients, and ‘borderline’ for a further 12.9%. Informants only reported lower than average levels of pro-social skills (I: d = -0.75, SR: d = -0.05), particularly for females (I: d = -0.89, SR: d = -0.37). The individuals themselves reported higher than average levels of hyperactivity (SR: d = 0.62) and a smaller effect was reported by informants (I: d = 0.32). Informants rated a moderate level of conduct problems (I: d = 0.52, SR: d = 0.05).

Scores for obsessive-compulsive symptoms (CY-BOCS-SR) were within the clinically significant range for obsessive-compulsive disorder in 56.4% of the sample. Scores were significantly correlated with duration of illness (Spearman’s correlation coefficient (*r*_s_) = 0.26, *p* <0.001), BN symptom severity (*r*_s_ = 0.34, *p* <0.001), ED clinical impairment (*r*_s_ = 0.51, *p* <0.001), depression (*r*_s_ = 0.42, *p* <0.001) and anxiety (*r*_s_ = 0.45, *p* <0.001), but not with BMI, lowest BMI or AN symptom severity.

### Diagnostic assessment of autism spectrum disorder

Compared with normative data, more AN females were assigned to the higher diagnostic probability bands of the DAWBA (10.9% AN versus 1.2% norms at level 2 (>3% likelihood of ASD) and 2.5% AN versus 0.3% norms at level 3 (>15% likelihood of ASD)).

Six individuals (4%, all female) were assigned a possible (n = 5) or definite (n = 1) diagnosis of ASD by the clinical rater. Clinical and psychometric features of these cases are displayed in Table 2. Informant-rated scores for total difficulties (d = 0.95) and impact (d = 1.47) were higher for this subgroup.

### Parental autism spectrum and obsessive-compulsive traits

Levels of autism spectrum and obsessive-compulsive traits in parents of adolescents with AN were within the normal range (Table 3). Only 2.2% of parents (n = 2 (3.1%) fathers, n = 2 (1.4%) mothers) scored within the clinically significant range suggestive of ASD. Fathers produced higher scores than mothers (t = -3.32, *p* = 0.001, d = 0.51). Scores for obsessive-compulsive traits (OCI-R) were within the clinically significant range for 7.9% of parents.

**Table 3:** **Parental traits**

	All parents			Parents of ASD subset only		
	**Mothers** **(N =** **143)**	**Fathers** **(N =** **64)**	**Total** **(N =** **207)**	**Mothers** **(N =** **6)**	**Fathers** **(N =** **6)**	**Total** **(N =** **12)**
AQ-10	1.95 (1.52)	2.81 (1.84)	2.20 (1.66)	1.80 (1.10)	2.67 (1.53)	2.13 (1.25)
OCI-R	8.85 (9.06)	7.50 (7.23)	8.47 (8.59)	14.00 (9.43)	6.33 (4.04)	11.13 (8.44)

Means and SD displayed; Abbreviations: ASD, Autism spectrum disorder; AQ-10, Autism Quotient; OCI-R, the Obsessive-Compulsive Inventory Revised.

There were no significant differences in autism spectrum traits (AQ-10) between parents of those assigned an ASD diagnosis compared with those who were not.

### Correlations between autism spectrum and obsessive-compulsive traits

Table 4 presents the correlations between autism spectrum (social aptitude and SDQ peer problems, and pro-social behaviors) and obsessive-compulsive traits (CY-BOCS-SR) in patients and in mothers and fathers (AQ-10 and OCI-R). Patient autism spectrum traits (social aptitude, peer difficulties, and pro-social behaviors) and obsessive-compulsive traits were not significantly correlated. Parental autism spectrum (AQ-10) and obsessive-compulsive traits (OCI-R) were significantly correlated for mothers only. Parental traits were not correlated with autism spectrum or obsessive-compulsive scores in patients.

**Table 4:** **Correlations between autism spectrum disorder and obsessive**-**compulsive disorder traits in female patients and their parents**

	Patient variables									Parent variables			
	1	2	3	4	5	6	7	8	9	10	11	12	13
1. Social aptitude	-												
2. Peer problems^a^	-0.27	-											
3. Peer problems^b^	-0.40**	0.60**	-										
4. Pro-social skills^a^	0.17	-0.35**	-0.15	-									
5. Pro-social skills^b^	0.58**	-0.18	-0.38**	0.18	-								
6. Emotion difficulties^a^	-0.19	0.38**	0.27	-0.17	-0.09	-							
7. Emotions difficulties^b^	-0.29*	0.23*	0.53**	0.03	-0.32**	0.34**	-						
8. CY-BOCS-SR	-0.05	0.16	0.20*	-0.13	-0.09	0.32*	0.18	-					
9. ASD Diagnostic Band	-0.45**	0.31*	0.40**	-0.13	-0.27*	0.32*	0.32**	0.19	-				
10. Mother AQ-10	-0.11	0.15	0.20	0.03	-0.01	0.18	0.05	0.06	0.19	-			
11. Father AQ-10	-0.13	0.32	0.33	0.22	-0.11	-0.06	0.17	-0.14	0.09	-0.23	-		
12. Mother OCI-R	-0.20	0.19	0.21	-0.14	-0.09	0.15	0.24	0.07	0.24	0.32**	0.14	-	
13. Father OCI-R	-0.35	0.25	0.29	0.05	-0.39	0.13	0.26	-0.02	-0.01	0.04	0.31	-0.01	-

Spearman’s correlation coefficient (*r*_s_) applying a Bonferroni-corrected alpha (α = 0.004); Abbreviations: AQ-10, Autism Quotient; ASD, Autism spectrum disorder; CY-BOCS-SR, Children’s Yale-Brown Obsessions and Compulsions Symptom Scale Revised; OCD, Obsessive-compulsive disorder; OCI-R, the Obsessive-Compulsive Inventory Revised.

^a^Strength and Difficulties Questionnaire by self-report.

^b^Strength and Difficulties Questionnaire by informant.

*p <0.003, **p <0.001.

## Discussion

The first aim of this study was to examine whether adolescents with AN have traits suggestive of ASD. We confirmed our first hypothesis in that we found that poor social aptitude skills and peer difficulties were more prevalent in the AN patients than in healthy population norms. We also found high co-morbid obsessive-compulsive traits in the patient group. We found that six (4%) patients met the diagnostic criteria for a possible or definite ASD. However, we failed to confirm our hypothesis that the parents of the patient group would have higher than normal levels of autism spectrum and obsessive-compulsive traits.

The prevalence of autism spectrum traits (low social aptitude (18.8%) and peer difficulties) is similar to the approximate 20% of the cohort of adolescent cases of AN who were thought to have social communication difficulties reported by Gillberg [1] and the 23% of children with restrictive eating problems who also screened positive for ASD [10]. It is lower than the 40% of adolescents fulfilling the Baron Cohen's criteria for the broad autistic phenotype [8]. The different populations, screening, and diagnostic procedures used in these studies may account for this variance.

The prevalence of diagnostically defined cases (4%) is lower than the proportion (23%) of adult patients with a severe and enduring ED who were considered to have ASD [36]. In part this may relate to the use of the DAWBA as a developmentally based diagnostic measure; however, given the poor prognosis of this group [37], it might be expected that a greater proportion of cases with ASD will be found in cohorts with a severe and enduring illness.

The six individuals with possible and definite ASD had a similar clinical profile to the group as a whole. However, informant ratings suggested that this subgroup had greater overall difficulties. However, given the small number, group comparisons are underpowered and these descriptors are therefore unreliable.

The problems in social functioning are consistent with the previous literature. Patients with EDs particularly those with AN retrospectively report social difficulties in childhood [38]. In a longitudinal study, social problems at age eight were strongly predictive of ED onset at age 14 [39]. Furthermore, a recent systematic review has documented the wide range of difficulties within the social cognition domain [40]. Although there may be ‘state’ effects of AN on neuropsychological functioning, the lack of association between social aptitude and current AN features is more indicative of a trait level of disturbance. The level of other forms of comorbidity in this group (obsessive-compulsive disorder traits, negative affect and some Attention Deficit/ Hyperactivity Disorder symptoms) is similar to that in the literature [41–43].

We did not find that the parents of patients with EDs had high levels of autism spectrum traits themselves. Parents of people with ASD have been found to have high autism quotient scores [44, 45], mainly in the social and communication domains [46, 47], suggesting that there is a broad familial ASD phenotype. However, this finding is less robust in mothers of people with ASD [48]. The small number of cases and the different instruments used makes the interpretation of possible cross-generational traits versus possible ‘state’ effects of AN difficult.

### Strengths and limitations

One of the strengths of this study was the use of the DAWBA as an assessment measure that has been widely employed in epidemiological studies of child and adolescent psychiatry, with healthy population norms available for comparison. Furthermore, because the DAWBA is framed developmentally and collects self-report and informant perspectives, it is less reliant on current symptoms or impairment (‘state’ effects of AN). Another strength of this study is the inclusion of both child and parent data. Nevertheless, several limitations must be noted; we used instruments that measured obsessive-compulsive disorder behaviors and it would have been preferable to have included instruments to measure traits associated with compulsivity, an over-controlled temperament, and obsessive-compulsive personality. Additionally, the internal consistency of the AQ-10 was poor and caution must be taken when interpreting these results. It would have been preferable to have also screened the participants themselves with the AQ-10. The possibility that the DAWBA is less diagnostically sensitive than an in-depth face-to-face clinician examination must also be considered. Given that the DAWBA assigned fewer AN and overall ED diagnoses in a sample of clinically diagnosed and treatment-seeking AN and EDNOS-AN patients, it is possible that the DAWBA provides a relatively conservative diagnostic tool. Furthermore, the patient previously diagnosed with ASD prior to entering the study was assigned a ‘probable’ (rather than ‘definite’) diagnosis using the DAWBA in the present study. It is therefore possible that the prevalence of ASD in AN may be higher than that reported in this study. Finally, the data presented do not represent all treatment-seeking patients, but only the group who consented to be involved in a study that involves their parents. It is possible that the sample is therefore biased towards patients with parents who were more actively involved in their child’s care.

### Clinical implications

We have found that a small number of adolescent AN cases are possibly comorbid with ASD. We have also found evidence of transdiagnostic traits such as social communication and emotional problems. These may be associated with ASD, the over-controlled temperament [49], and obsessive-compulsive personality [50] in adolescents with EDs. Treatment adaptations to target these difficulties may be beneficial.

## Conclusions

Approximately one fifth of adolescents with AN have obsessive-compulsive traits and problems in peer relationships and social functioning. A much smaller proportion (4%) fulfil the diagnostic criteria for a probable ASD. It will be of interest in the future to establish the prognosis of this subgroup and whether these traits moderate the response to standard treatment, as suggested by Crane *et al*. [51].

## Abbreviations

AN: Anorexia Nervosa
AQ: Autism Quotient
ASD: Autism Spectrum Disorder
BMI: Body Mass Index
BN: Bulimia Nervosa
CIA: Clinical Impairment Assessment
CY-BOCS-SR: Children’s Yale-Brown Obsessive-Compulsive Scale Self-Report
DASS: Depression Anxiety Stress Scales
DAWBA: Development and Well-being Assessment
DSM-IV: Diagnostic and Statistical Manual of Mental Disorders
ED: Eating Disorder
I: Informant
ICD-10: International Statistical Classification of Diseases and Related Health Problems
OCD: Obsessive-Compulsive Disorder
OCI-R: Obsessive-Compulsive Inventory Revised
SAS: Social Aptitude Scale
SD: Standard Deviation
SDQ: Strengths and Difficulties Questionnaire
SEED: Short Examination of Eating Disorders
SR: Self-Report.
